# Early psychological responses of children and caregivers in the immediate aftermath of release from war captivity

**DOI:** 10.3389/fpsyg.2025.1588422

**Published:** 2025-05-23

**Authors:** Maya Fennig, Avigal Snir, Maayan Shorer, Efrat Bron Harlev, Silvana Fennig

**Affiliations:** ^1^The Bob Shapell School of Social Work, Tel Aviv University, Tel Aviv, Israel; ^2^School of Behavioral Sciences, Academic College Tel Aviv-Yaffo, Tel Aviv, Israel; ^3^Psychological Medicine Department, Schneider Children's Medical Center, Petach Tikva, Israel; ^4^Clinical Psychology Department, Faculty of Social & Community Sciences, Ruppin Academic Center, Emek Hefer, Israel; ^5^Gray Faculty of Medical & Health Sciences, Tel Aviv University, Tel Aviv, Israel

**Keywords:** children, trauma, war, Israel, conflict, mental health, adolescents, psychological intervention

## Abstract

**Background:**

Captivity—particularly the captivity of children—is one of the most extreme violations of civilian rights in armed conflict. Despite this, most research on war captivity has focused on adult soldiers, leaving largely unexplored the unique psychological reactions of children subjected to such trauma.

**Objective:**

This study aims to (1) describe children and caregivers' early psychological responses immediately following their release from captivity in the Israeli-Hamas war and (2) examine the clinical interventions used to manage these reactions.

**Methods:**

This qualitative study analyzed the psychological reactions of children and their caregivers (*N* = 26) who were released from captivity and received care at Schneider Children's Medical Centre of Israel. Data collection methods included a review of medical files and in-depth interviews with practitioners (*n* = 37), including social workers, psychologists, psychiatrists, nurses, and pediatricians, who provided health- and psychosocial care.

**Results:**

Psychological responses were influenced by developmental stage and captivity context. Among young children (2–11 years), predominant reactions included excessive worry, repetitive questioning, traumatic reenactment (e.g., through play), separation anxiety, hypervigilance, anger outbursts, low frustration tolerance, and sleep disturbances. Adolescents (12–18 years) primarily exhibited avoidance, hyperarousal, sleep disturbances, and excessive sharing of traumatic experiences. Caregivers (19–80 years) displayed reactions similar to those of adolescents but also demonstrated dissociative reactions. Interventions were based on the principles of the Psychological First Aid (PFA) model and tailored to the specific reactions of children and caregivers.

Conclusions: This study is the first to provide qualitative data on the psychological responses of children and caregivers following war captivity. The findings highlight the need for a family-oriented approach to mental health interventions, particularly for supporting young children and their caregivers. Training practitioners to recognize developmentally specific reactions in the immediate aftermath of captivity is critical for effective assessment, care, and psychopathology prevention. The study concludes with recommendations for improving practice and policy to address this severe and complex violation of children's rights.

## 1 Introduction

The nature of modern warfare is changing, with a growing number of civilians being harmed, whether caught in the crossfire or deliberately targeted. Among the most alarming developments is the mass abduction of civilians, particularly children, which has emerged as a defining feature of contemporary armed conflict. Abduction and captivity in the context of armed conflict are among the most severe and complex violations of children's rights. These rights— including protection from violence, the right to life and development, access to healthcare, family care, and protection from parental separation—are enshrined in Articles 6, 19, 24, and 37 of the United Nations Convention on the Rights of the Child and international humanitarian law.

Between 2005 and 2022, more than 32,500 children were verified as abducted by parties involved in conflicts, with an estimated 2,500 to 4,000 children abducted each year (United Nations Children's Fund (UNICEF), 2023). Child abduction in armed conflict is one of the six grave violations identified and condemned by the UN Security Council, and it frequently precedes other serious abuses such as sexual violence and forced recruitment into armed groups (United Nations Children's Fund (UNICEF), 2022). The United Nations Children's Fund (UNICEF) (2022) defines child abduction in armed conflict as the removal, seizure, capture, apprehension, taking or enforced disappearance of a child either temporarily or permanently, including for the purpose of any form of exploitation of the child. […] [T]he abduction must be perpetrated by a party to conflict in the context of and be associated with an armed conflict. (p. 14).

Abduction and captivity in war are widely recognized as exceptionally traumatic and pathogenic experiences (Ursano and Benedek, 2003; Solomon et al., 2008). Hostages endure prolonged, repeated exposure to traumatic stressors, including a constant state of fear of torture or death, forced social isolation, and total dependence on their captors, who often intentionally exacerbate their psychological distress (Zerach et al., 2019). In addition to the deprivation of basic physiological needs such as food, water, and sleep, war-captivity trauma is uniquely amplified by its protracted duration and the interpersonal power dynamics at play (Herman, 1992).

Most research on war captivity centers on adult soldiers (Solomon et al., 2008; Zerach et al., 2019), with few studies exploring the distinct experiences and psychological responses of children who experience captivity during armed conflict (Gossmann et al., 2024; Terr, 1979). Emerging evidence indicates that war-abducted children are at risk of developing mental health disorders due to continuous traumatic stress exposure (Gossmann et al., 2024). These disorders include post-traumatic stress symptoms, internalizing and externalizing problems, suicidality, major depressive disorder, aggression, and generalized anxiety disorder (Gossmann et al., 2024; Betancourt et al., 2013; Pham et al., 2009).

In a 2015 study, Winkler et al. evaluated clinically significant symptoms of posttraumatic stress disorder (PTSD) in formerly abducted Ugandan youth, using the Posttraumatic Diagnostic Scale. The authors reported that 32% exhibited clinically significant posttraumatic stress reactions, compared to 12% among non-abducted peers. Similarly, a study of former abducted female child soldiers forcibly recruited during the M23 insurgency (2012–2014) in Eastern DRC found that participants had high levels of PTSD and depression two and a half years after their escape or release (Robjant et al., 2019). Both quantitative and qualitative studies indicate that upon reintegration, a large proportion of war-abducted children and adolescents experience difficulties with adjusting to civilian life and face relationship challenges (Pham et al., 2009; Denov, 2010).

Despite this population's acute vulnerability, emerging scientific research is focused on the *long-term* mental health effects of abduction and war captivity, with assessments often conducted months or years later (Betancourt et al., 2010, 2020; Pfeiffer and Elbert, 2011). To date, few studies have assessed children's early psychological responses in the *immediate aftermath* of their release. Additionally, most literature in this field is based on studies of children associated with armed forces and armed groups (Betancourt et al., 2010; Winkler et al., 2015; Pham et al., 2009). However, findings about child soldiers may not be directly applicable to other war-abducted children, as their experiences differ significantly. Unlike other war-abducted children, child soldiers often play a dual role as both victims and perpetrators of violence, a factor that shapes their psychological needs and trauma in distinct ways (Fennig and Denov, 2024; Betancourt et al., 2020). Moreover, in many instances, symptom data are collected using checklists, which, while useful, fall short of capturing the full spectrum of symptoms. Indeed, since they are seldom validated by the local population, these tools can fail to account for the nuanced and complex emotional and psychological responses that children experience in the critical period immediately following their release from captivity (Gossmann et al., 2024; Betancourt et al., 2013).

If one does not know what symptoms to expect, how can one design effective early interventions? To our knowledge, no clinical guidelines exist for assessing and caring for children and adolescents immediately following war captivity. This gap is worrisome, as early symptom detection and intervention in the initial hours, days, or weeks after release from captivity can significantly alleviate acute stress and help prevent long-term psychological disorders (Birur et al., 2017).

To address this knowledge gap, this study examines data from medical records and in-depth interviews with practitioners who provided medical and psychosocial care to children and caregivers in Israel following their release from captivity. Recognizing that symptom expression and clinical interventions reciprocally inform each other, the study aims to (1) describe children's early psychological responses and symptom expression in the immediate aftermath of release from captivity in the Israeli-Hamas war and (2) examine the interventions used by clinicians to manage these symptoms.

## 2 Methods

### 2.2 Context and setting

In Israel, the mental health effects of war-related child abductions are of particular concern. On October 7, 2023, Hamas and other Palestinian militant groups launched a major assault on Israel from the Gaza Strip. This attack resulted in approximately 1,200 fatalities and over 5,400 injuries, predominantly among civilians, along with the abduction of 253 hostages. In response, Israel initiated an extensive military campaign in Gaza which, according to a study published in The Lancet, had resulted in more than 64, 200 Palestinian deaths, over 106,000 injuries, and the displacement of the majority of Gaza's population, creating a severe humanitarian crisis (Jamaluddine et al., 2025; United Nations Office for the Coordinated Humanitarian Affairs (OCHA), 2024).

On October 7, children were intentionally targeted for abduction. Thirty-six Israeli children aged 8 months to 18 years were taken into Gaza, 10 of whom were abducted alone, without family members. The children were held in changing locations, including underground tunnels, guarded by armed captors, exposed to bombings, deprived of food, and subjected to various forms of physical and psychological violence (Rosman et al., 2023). In some cases, captors intentionally separated children from their parents. In November 2023, after nearly 2 months in captivity, 105 hostages were released, including thirty-four children. Upon their release, thirteen children were forced to leave a parent behind in captivity (Israeli National Council for the Child, 2023). The released children were immediately transported to Israeli hospitals, where they received comprehensive medical care, including psychological assistance (Israel Ministry of Health, 2023).

This exploratory study was part of a larger research project evaluating the implementation of PFA-CC, an intervention delivered in hospital settings by a multidisciplinary team of practitioners to children and adolescents (aged 2.5–18 years) and their caregivers immediately following their release from war captivity. Conducted over a 4-month period (December 2023–March 2024), the study was a partnership between Tel Aviv University and Schneider Children's Medical Centre of Israel. Results from the evaluation of the PFA-CC intervention are reported elsewhere {authors own}. Despite the promising utility of the PFA-CC protocol, its flexible and modular nature provides only broad guidelines for treatment, without specifying how therapeutic techniques should tailored to particular symptoms or age groups. Thus, this companion paper provides detailed descriptions of children's psychological symptoms in the immediate aftermath of their release from war-captivity and specifies the clinical interventions used to mitigate them.

### 2.2 Study sample

Table 1 displays the characteristics of the sample of children and their caregivers (*N* = 26) who had been abducted in the context of war and subsequently received treatment at Schneider Children's Medical Centre of Israel, where the study was conducted. The sample comprised 19 children (aged 2.5–18; 11 females and 8 males) and 7 caregivers (aged 30–78; all females). They received treatment immediately following their release from captivity, with hospitalizations lasting from 2 to 8 days. All children and caregivers identified as Jewish and were citizens of Israel. More than half of them had witnessed the death of a loved one (*n* = 14) or of a community member (*n* = 20). Nearly all (*n* = 23) had suffered deprivation of basic needs (e.g., sleeping, eating, hygiene), while 8 had suffered physical violence. Several children (*n* = 8) had been held in captivity without their parents.

**Table 1:** Characteristics of the intervention's target population, according to the medical team's reports (*N* = 26): age, gender, and exposure to potentially traumatic experiences.

**Variable**	**Frequencies**	**Mean**	**SD**	**Range**
Gender	Female – 18 Male – 8			
Age	Under 18–19	9.47	5.23	2.5 – 18
	Over 18–7	47.28	14.14	30 – 78
**Exposure to traumatic experiences during captivity:**				
Witnessed someone close getting killed	Yes – 14 No – 10 Unknown – 2			
Witnessed a stranger getting killed	Yes – 20 No – 1 Unknown – 5			
Witnessed someone close being injured	Yes – 25 No – 1			
Witnessed a stranger being injured	Yes – 22 No – 0 Unknown – 4			
For children: Was isolated from both his/her parents	Yes – 8 No – 11			
Experienced verbal violence	Yes – 13 No – 2 Unknown – 11			
Experienced physical violence	Yes – 8 No – 16 Unknown – 2			
Experienced hunger	Yes – 22 No – 2 Unknown – 2			
Was deprived of basic needs (sleep and hygiene)	Yes – 23 No – 1 Unknown – 2			
Moved from place to place	Yes – 25 No – 1			
Experienced sexual abuse	Yes – 1 No – 25			

The study sample comprised a subset of 37 practitioners employed at Schneider Children's Medical Centre of Israel, including social workers (*n* = 11), psychologists (*n* = 10), a child psychiatrist (*n* = 1), nurses (*n* = 9), pediatricians (*n* = 4), and senior directors (*n* = 2). These professionals delivered the PFA-based intervention. Initially, 48 practitioners were identified by department directors. Of these, 11 practitioners who had expressed interest in participating in the study were unavailable for interviews during the study period due to job constraints. The final sample thus included 34 women and 3 men, aged 33–68 (M = 49, SD = 9.4). All participants identified as Jewish and were Israeli citizens. All participants held an academic degree (59% have a Master's degree), and their professional experience ranged from 6 to 40 years. Most (*n* = 34) had a prior experience working with traumatized children, while 3 reported no such experience.

### 2.3. Ethical considerations

All procedures were reviewed and approved by the Institutional Review Board of Tel Aviv University and the Institutional Review Board of Schneider Children's Medical Centre of Israel. Given the study's sensitive nature and the high-profile status of the participants due to media coverage, the research team implemented additional measures to ensure confidentiality and uphold ethical standards. Although the children and adolescents were not directly interviewed, their informed consent, along with that of their families, was obtained. Every effort was made to safeguard their identities, including the use of pseudonyms and the careful omission or blurring of any potentially identifying details.

### 2.4. Data collection and analysis

This qualitative study utilized multiple data sources to provide rich, complementary insights. First, in-depth interviews were conducted with practitioners. These interviews, which lasted between 1 and 2 h, were carried out face-to-face by the first author and a research assistant (a clinical psychologist). Both interviewers were external to the implementation of the PFA-CC intervention.

The interview questions were broad and exploratory, canvassing clinicians' observations of symptom expression in the children and caregivers they treated. In addition, practitioners were asked a limited number of close-ended questions. For example, they were asked to review a list of emotional and behavioral responses commonly observed in individuals following trauma and indicate whether they had observed each reaction in their patients. This list was derived from validated measures, including the Pediatric Emotional Distress Scale (PEDS; Saylor et al., 1999) for young children's emotional distress, the Children and Adolescent Psychological Distress Scale (CAPDS-10; De Stefano et al., 2022), and the acute stress reactions outlined in the *Diagnostic and Statistical Manual of Mental Disorders* (American Psychiatric Association, 2013). Participants were also asked to report any symptoms not included on the list, as well as to describe their decision-making processes and approaches to symptom management.

Additionally, the research team cross-validated and expanded upon the reported reactions and interventions in the interviews by reviewing the medical files of children and caregivers. Each medical file contained records by the multidisciplinary team who treated the child, documenting their observations on physical and mental health status, along with details of any interventions performed.

Analysis of the data was conducted in three stages. In the first stage, all available data on each child and caregiver, including interviews and medical records, were compiled. Since each patient was treated by a multidisciplinary team (pediatrician, nurse, psychologist, and social worker), the team cross-referenced interviews from all four practitioners. A reaction was considered evident only if it was documented in the medical record and reported by at least three of the four team members.

In the second stage, reactions were categorized by age group. Only reactions reported in at least 80% of children and caregivers within each age group were considered significant. In the third stage, a thematic analysis of practitioner interview transcripts was conducted, integrating both deductive coding (applying predefined categories based on reactions identified in Stage 1) and inductive coding (allowing themes to emerge organically from the data). Coding was aligned with the study's research objectives—identifying early reactions and associated interventions. Following the conventions of thematic analysis (Braun and Clarke, 2006), patterns, contradictions, and variations across the interviews and medical records were systematically examined; themes were thus refined through an iterative process.

## 3 Results

Analysis of the data revealed that, in the immediate aftermath of release, none of the observed reactions severely impaired normal functioning or required emergency interventions. The initial reactions were fluid, often diminishing over the course of the children and caregivers' hospital stay. Notably, the reactions and associated psychological interventions varied significantly across age groups, namely young children (2–11 years old, *n* = 12), adolescents (12–18 years old, *n* = 7), and caregivers (19–80 years old, *n* = 7). Practitioners' evaluations of early response symptoms among children and caregivers freed from captivity are presented in Table 2, categorized by age group. Additionally, findings from the qualitative analysis include clinical vignettes shared during interviews. These vignettes provide deeper insight into the most frequently observed symptoms and corresponding clinical interventions.

**Table 2:** Practitioners' evaluations of early response reactions among children and caregivers freed from captivity (reaction considered significant only if present in 80% of cases in age group).

**Children (2–11years)**	
1	Worry and repeated questioning
2	Traumatic reenactment (play)
3	Separation anxiety
4	Vigilance
5	Burst of anger (low frustration tolerance)
6	Sleep disturbances
7	Nightmares
**Adolescents (12–18 years)**	
1	Avoidance
2	Vigilance and agitation
3	Sleep disturbances
4	Intrusive thoughts
**Adults (30–78 years)**	
1	Disconnection and disorientation
2	Traumatic reenactment (telling the story)
3	Sleep disturbances
4	Agitation
5	Thoughts of institutional betrayal and warm feelings toward captors

**Theme 1: Reactions and psychological interventions among young children, aged 2.5–11**.

Theme 1.1: Worry and repeated questioning

The most common early reactions among young children were worry and repeated questioning. Although these young children were no longer in captivity or imminent danger, all expressed fears and concerns about their safety, both directly and indirectly, in the immediate aftermath of their release. For example, Ron, a 4-year-old boy, expressed his worry that he would be returned to Gaza through repeated questioning about his surroundings. His therapist recalled:

I joined him in a game he was playing, and I started creating a connection with him through the game, and what was very clear was that he was mostly very confused between Gaza and Israel. Between the soldiers here and the soldiers there. He was abducted by people who were wearing uniforms – for a four-year-old boy, this looked like Israeli soldiers. It was very confusing. It was tough for him to understand – Am I now in Israel, am I going back to Gaza? (Psychologist, E8).

At first glance, Ron's behavior might be misinterpreted as confusion. However, a closer reading of his repeated questioning reveals that Ron was not disoriented, but rather attempting to differentiate between “good” adults (family, Israeli soldiers) and “bad” adults (kidnappers)—a pressing concern given that certain elements in the hospital environment (e.g., military uniforms) evoked fear and the corresponding worry that he might still be in danger.

Intervention: to help Ron differentiate between events in captivity and in Israel, his therapist used concrete objects in the hospital and its surroundings, such as his favorite food, to symbolize safety. The therapist shares how this strategy provided reassurance and emotional adjustment:

There was a lot of work at first on differentiating between what happened and the current environment, even using food. For instance, when he arrived, he really wanted pretzels so when we brought him pretzels I told him: ‘In Gaza, there were no pretzels, pretzels are a food that we have only in Israel.' There was even a moment when we were looking out the window and I said: ‘These are buildings we have in Israel, this is not how Gaza looks like.' Like really making that differentiation (Psychologist, E8).

Theme 1.2: Traumatic reenactment (Creates stories/traumatic play)

Another frequently observed reaction among children while in the hospital was the reenactment of captivity themes, either through behavior or play. For example, one of the therapists reported that 2.5-year-old Dana, in the first days after her release, insisted on wearing the same clothes that she had worn throughout captivity, despite her mother's attempts to change them:

In captivity, she was used to wearing diapers, a tank top, and socks. Not children's socks, these were adult socks that came up to their knees. And that's how she came back with the diaper and the socks. When she got here, we provided her with new children's clothes. and her mother tried to get her dressed but Dana refused and started crying (Psychologist, E6).

Intervention: after consulting with the team, the therapist decided to adopt a non-intrusive approach that respected the little girl's need to maintain habitual behavior from captivity.

And the mother didn't know what to do with all this. At first, she thought that it was not acceptable [wearing the same clothes as in captivity]. Then we [the practitioners] consulted and concluded that we shouldn't push her to make this change, that we need to respect her wishes. (Psychologist, E6)

The therapist then provided psychoeducation to the mother, explaining that the reenactment was not a sign of pathological anxiety but rather an adaptive coping mechanism that would gradually diminish on its own:

So, I met with the mother and explained to her the rationale. She quickly understood and spoke with her daughter and the whole team started running around to buy her socks. (Psychologist, E6)

Another common manifestation of traumatic reenactment among young children was aggressive play. One therapist observed Noya, a 3-year-old-girl, who reenacted aspects of captivity, possibly exhibiting identification with the aggressor or anger at not being protected:

In this game she was exhibiting this aggression toward me, there was an alligator, and the alligator came to eat me. I was just there; I didn't continue and provide her with an interpretation. It was very delicate—how much to provide a therapeutic response and in what way.

Intervention: in response, the therapist let Noya freely express her affect during play. She did not discourage her from expressing aggressive themes, but instead demonstrated that she could tolerate it in a safe environment, as if saying “I'm here to protect you”:

So I reacted, I mean I reacted in a way that was not frightened by it and a bit frightened as a joke. I mean it was still important for me to decrease the aggression and for her to feel my resiliency. For her to see that I notice her aggression and that I'm not frightened by it or ignoring it by continuing to play (Psychologist, E10)

Theme 1.3: Hypervigilance and separation anxiety:

Traumatic reenactment often co-occurred with other reactions, particularly separation anxiety and hypervigilance, with all young children expressing constant arousal and difficulty regulating their behaviors. One example of this overlap is Noah, a 4-year-old child who had been held captive with his mother and siblings. During his hospital stay, he displayed separation anxiety vis-à-vis his mother as well as emotional turbulence, including bursts of intense emotion. He also engaged in ritualistic eating, a possible sign of traumatic reenactment, and developmental regression. His therapist explains:

The youngest child - I think he had the hardest time there, in captivity – he was emotionally turbulent, I mean he would cry a lot. He was very needy and clung to his mother. He would cry hard when she needed to leave him for a bit - to go to interrogation or to take a shower. He also had a hard time eating. The father was shaken by the way he ate. He would crumble the food and store it in his mouth, it was really very hard to witness. There was also an issue with going to the bathroom. He repeatedly passed urine and feces in his underwear instead of the toilet. Although the mother said that in captivity it didn't happen. (Psychologist, Female, E3)

Intervention: the therapist provided psychoeducation to the parents, normalizing the young boy's behaviors by connecting them to his basic temperament, which was sensitive and reactive to changes. She also encouraged a gradual approach to resuming normal eating patterns:

Parents wanted to know how to respond to his behaviors, they were most concerned with his eating. We talked about slowly exposing him to normal routine meals and encouraging him patiently to swallow the food in order to be able to go back to playing. After 2 days or so, he went back to normal eating. By the time the family left the ward all these symptoms were not present. (Psychologist, Female, E3)

Another example of hypervigilance and separation anxiety was observed in Sara, a 3-year-old who had been held captive with her mother. While in the hospital, Sara was very reactive to her mothers' feelings and behaviors, and was constantly worried and fearful about being separated from her mother:

She noticed every nuance, and the moment the mother's state, for example, was not so good she would immediately react to it…. She was very alert: ‘Where is Mommy?' ‘How will Mommy react?' I remember I was with her one day after the mother left for the security cabinet and came back. I was with her in the morning, and she was a lot more needy. This was the first time they were apart after two months. (Psychologist, Female, E10)

Intervention: the therapist helped Sara understand her fear and separation anxiety in a simple, concrete way:

So I shared with her and with the mother: “Is it possible that you are checking if Mommy is here, because you are not sure that Mommy left and came back? And suddenly this was different for you and you want to see that she is not leaving again?” (Psychologist, Female, E10)

The therapist noted that this brief but direct intervention calmed Sara down and reduced her anxiety: “She was able to simply take this small interpretation and calm down and return to her game” (Psychologist, Female, E10)

**Theme 2: Reactions and psychological interventions among children and adolescents, aged 12-18**.

Theme 2.1: Avoidance

Among adolescents, avoidance was a common response following their release from captivity. Many had been held alone, separated from their caregivers, and upon their return, they often actively distanced themselves from unpleasant emotions and memories of their trauma. A stark example is 17-year-old Ron, as described by his therapist: “He was very afraid of being alone. He said, 'I have thought about it a bit, and I have some requests. I need someone to sleep with me and take a shower with me.” (Psychologist, Female, E9).

Nearly all the adolescents were avoidant, preferring to delay exposure to information concerning October 7. However, complete avoidance was frequently impossible, leading to unplanned exposures to traumatic triggers or reminders, such as learning about the deaths of family members and friends. For instance, when 16-year-old Ben was suddenly exposed to such information, he experienced extreme agitation and distress:

When he first got to the hospital his approach was “I don't want to know what happened in the Kibbutz”... At night I discovered that he took the phone and independently found out everything that happened (on Oct 7) and he became very agitated. (Psychologist, Male, E5)

In cases of avoidance followed by overexposure, therapists guided parents and caregivers in delivering difficult news and in slowly adjusting to post-captivity reality. Therapists followed principles of gradual exposure, organizing the information in such a way as to help maintain structure and calm throughout the conversations. Therapists unanimously emphasized the importance of creating a safe and quiet time for delivering the difficult news, as opposed to delivering information “along the way.” For example, when Ben became agitated after being exposed to all the difficult news about his friends and loved ones, his therapist set up a structured session with him and his parents:

I told them ‘Wait let's do this organized'. I sat them down in a room together with the son and I said Let's go, talk.. Don't be scared, let's touch it, let's put things on the table. You (Ben) can go ahead and ask all the things you want to know; you (the parents) tell him.' Then this session was created where the parents were telling him, and I was mediating. And slowly, slowly he started asking and he got information – how many people were abducted, how many died, what happened, names of people. The parents sometimes looked at me, asking if they should say something or not. So I was mostly there to provide support”. (Psychologist, Male, E5)

Therapists reported that these conversations, although very difficult and emotional, were necessary for beginning the adjustment process. Following a consultation with his psychiatrist, Ben was administered medication (Klonex) to calm him down and help him sleep: “I think when that was over, we could start the grieving process – but first, it was important to face reality and calm down.” (Psychologist, Male, E5)

Avoidance persisted for many of the adolescents upon discharge. In an attempt to avoid or distance themselves from distressing memories, thoughts, or feelings, some expressed hesitation about returning to their communities and former activities. Certain families even sought to delay discharge entirely, underscoring the complex challenges of transitioning out of the ward and back into daily life after captivity. The struggle was particularly evident for 16-year-old Ranny, whose therapist described the family's reluctance to leave the hospital:

The family didn't really want to be discharged. They asked us to find them a hotel. They weren't ready to go home… And Ranny didn't want to go back to anything; to school, to basketball, to anything. It all seemed to him insignificant. (Psychologist, Female, E11)

Practitioners validated these fears about leaving the ward, helping children and their families think through the reintegration process to regain functioning, as well as providing preventive education about post-traumatic disorders and other potential symptoms that might appear later on. Ranny's therapist described her psycho-education intervention, which focused on gradual re-exposure to routine:

I emphasized how important it is to go back to a routine, it even shouldn't be all at once, but he should choose something, little things to go back to gradually. After he shared that he didn't want to go back to his hometown I spoke to him about avoidance that can become expanded and generalized.” (Psychologist, Female E11)

Theme 2.2: Hyperarousal

Some adolescents exhibited hyperarousal reactions, including sleep disturbances, an increased startle response, hypervigilance, and irritability. For example, one participant reported that for 17-year-old Ron, auditory stimuli (such as the closing of doors in the hospital ward), caused an exaggerated startle response:

He also said that the closing of doors would remind him…In the tunnels [where he was kept during captivity] there were these big metal doors, so it would really make him jump (Social Worker, Female, A11)

Another mental health practitioner observed a similar reaction in Shira, the adolescent girl she treated. Physical touch, even from her brother's hand, provoked a strong reaction:

She was very startled when her brother touched her on the leg by accident, she jumped. She said to him ‘What? No one touches me without asking first.' It was clear that there was something very traumatic there. (Social Worker, Female, A3)

Theme 2.3: Sleep disturbance

Adolescents found nighttime particularly trying, reporting nightmares, fear, intrusive thoughts, and difficulty both falling asleep and maintaining sleep continuity. One social worker described an adolescent boy's nighttime distress:

My first experience of him was that he was in a state of shock, his eyes were like he was in a dream, like glass, you look at him and there is no eye contact. At first, he hardly spoke also the entire period he didn't cry. He just had this frozen look in his eyes. He didn't want to be alone in any moment. When he took a shower he asked for the presence of his mother, to be next to the shower not inside. During the nights he had trouble falling asleep and it was then that he wanted to talk a lot about captivity, with his mother, with his girlfriend and with us. (Social Worker, Female, A10)

Although vigilance, fear, and sleep disturbances were to be expected in these circumstances, participants intervened in various ways - through conversations, caregiver guidance, and, in some cases, medication. The goal was to enhance feelings of calm and safety, allowing the body to rest, recover, and gradually return to normal daily routines.

**Theme 3: Reactions and psychological interventions among caregivers (6 mothers and 1 grandmother), aged 19–80**.

In captivity, adult caregivers made a conscious effort to protect their young children and to instill in them a sense of safety by regulating their own emotions and suppressing expressions of their own fear or anxiety. One psychologist explains this effort by a mother:

She constantly thought about her daughter. She created within that space, which was so intolerable and so chaotic, daily routine. She knew how to communicate to her daughter things in a way that she felt safe, although there was no safety. (Psychologist, E10).

Upon return, however, caregivers exhibited a range of symptoms. Below are the most prevalent ones.

Theme 3.1: Dissociation

Some of the caregivers displayed disconnection from their surroundings, a cold or indifferent affect, and a sense of altered reality. In the case of Chaya, a mother freed from captivity with her child, dissociation helped her to cope with the agony of realizing that another family member would not be released in the hostage deal and would remain in captivity. Her therapist recalls:

There was one day that was extremely difficult. It was when the ceasefire ended, the fighting resumed, and the release of the hostages stopped. She (the mother) completely collapsed. When I came in the morning, she was catatonic, and her daughter was sitting next to her watching a movie. (Psychologist, Female, E10)

Intervention: the therapist attuned her approach to match Chaya's pace and readiness to share her traumatic experiences. By relieving her of the “protector role” and momentarily taking on the responsibility of caring for her child, the therapist communicated to Chaya that she was now in a safe and supportive environment. The therapeutic context created a space in which she felt “permitted” to express her emotional pain:

I think we were able to help her a bit to process this incomprehensible thing…I told her “Here you can rest. It's okay, there are a lot of people here and they are taking care of her (her child).” And that gave her permission to be in touch with the unbearable experience of terror and pain that she was in. And it gave her permission, in a way, to let go and trust others, and let her daughter trust others. (E10)

Theme 3.2: Extensive sharing of traumatic material

All caregivers engaged in extensive sharing of their trauma stories immediately upon return. They immediately provided highly graphic, detailed accounts of their experiences on October 7 and during captivity. This extensive sharing is illustrated in the case of Noga, who repeatedly recounted her traumatic story, in great detail, to both family members and the healthcare personnel:

She just started talking endlessly and it was like a flood of words. It was like she wanted to spill everything, and she jumped from this thing to that thing, because you know, she was trying to grasp it all. She also became tired of talking so much. She became hoarse when speaking. I mean she spoke a lot a lot a lot (Social worker, A4)

In line with the PFA-CC treatment protocol, Noga's therapist refrained from exploring traumatic memories in-depth, as doing so at this early stage of the intervention could cause flooding and intensify intrusive symptoms. She asked short, structured questions related to captivity, with the intention of shutting down Noga's automatic, emotionally loaded reactions to instead facilitate cognitive reorganization:

And what I did was help her, in a very gentle way, with the temporal order… help her organize the story. She was talking about what happened in captivity and jumping from day to day, so I helped her establish the timeline of what happened and when it happened (Social worker, A4)

It is important to note that this urge to share the traumatic story took many therapists by surprise. They had initially assumed caregivers would prefer to explore their trauma at more advanced stages of the recovery process, rather than immediately after release:

We sat for a lot of hours. She wanted to talk and share her story, and I was not prepared. I thought we won't even reach this point, that it won't happen in the hospital, only later (Psychologist, E2)

## 4 Discussion

This qualitative study of children, adolescents and their caregivers released from war captivity reveals that psychological responses in the immediate aftermath of release are multifaceted and shaped by both developmental stage and captivity circumstances (e.g., whether they were held as a family or separately). These reactions were dynamic and gradually subsided throughout hospitalization. None of the observed reactions severely impaired normal functioning or required emergency interventions. Interventions were based on the principles of the Psychological First Aid (PFA) model—safety, calm, self-efficacy, connectedness and hope—and were tailored to the needs and responses of each age group: children, adolescents, and adult caregivers (Hobfoll et al., 2007).

### 4.1 Young children

Among young children, the most prominent reactions included worry, repeated questioning, and traumatic reenactment. These reactions were often accompanied by other symptoms, particularly separation anxiety, hypervigilance, and behavioral dysregulation (manifesting as anger outbursts, low frustration tolerance, and sleep disturbances). Such reactions are recognized in the scientific literature as hallmarks of the acute post-disaster phase among young children (Moner et al., 2022).

A notable reaction in this age group was traumatic reenactment, which often involved the persistence of behaviors and rituals established during captivity. For instance, young children frequently maintained routines related to food consumption, eating patterns, and clothing preferences after release. When working with caregivers, practitioners should acknowledge that these behaviors may seem concerning or maladaptive. Nonetheless, caregivers should be reassured that such reactions are in fact typical in the acute recovery phase. Allowing children to temporarily continue these rituals can foster a sense of security, with the expectation that they will gradually diminish as the child transitions to the post-captivity environment.

Notably, young children may be more vulnerable than older children to the lasting negative consequences of adversity (Atazadeh et al., 2019; Dunn et al., 2017). This heightened vulnerability may be linked to the rapid development of brain structures during early childhood as well as young children's strong dependence on caregivers (Lyons-Ruth et al., 2017; Moner et al., 2022). Consequently, clinical attention to this age group is particularly necessary. In this study's sample, the majority of young children had been held in captivity with a caregiver (a parent or another adult), a fact which may help explain the relatively low levels of acute maladaptive reactions and their overall adaptive functioning in the immediate release phase. Studies on post-disaster adjustment among young children suggest that their ability to buffer stress is strongly influenced by the caregiver and overall social support system (Scheeringa and Zeanah, 2001).

Interventions for young children included both direct child-centered approaches as well as caregiver-mediated interventions. The latter included support and guidance, psychoeducation on common trauma reactions, and assisting caregivers in re-establishing daily routines for their children such as eating, playing, and sleeping. Child-centered interventions included providing a safe space for processing traumatic experiences through behavioral reenactment/play and non-intrusive reflective listening.

Practitioners also helped children differentiate between the unsafe captivity environment and the current safe reality (e.g., “The soldiers who abducted you were threatening, but not all adults in uniform are,” or “That night was unsafe, but not all nights are unsafe”). Consistent with developmental considerations, these interventions prioritized concrete, tangible aspects of experience (e.g., soldiers' clothing, types of food) over abstract concepts. At this initial stage, cognitive interventions aimed to promote organization and clarity rather than delve into the emotional processing of the traumatic experience. As such, interventions were kept brief and straightforward, designed to enhance cognitive structure and prevent overwhelming emotional responses (such as flooding) (Farchi et al., 2018).

### 4.2 Adolescents

Adolescents primarily exhibited symptoms of avoidance, hyperarousal, sleep disturbances, and excessive sharing of traumatic material. These reactions align closely with previous studies assessing symptoms and mental health responses after terrorist attacks. However, unlike prior research, we did not observe somatic complaints or dissociative symptomatology (Chauvelin et al., 2019; Ceri et al., 2016).

Avoidance manifested as fear of enclosed spaces (e.g., bathrooms or showers), fear of sleeping alone, and reluctance to leave the unit upon discharge. Its prominence in our sample may be attributed to the fact that nearly all adolescents had been held captive without their families. The cumulative trauma experienced on October 7 (prior to abduction), combined with prolonged social isolation during captivity, may have disrupted adolescents' ability to perceive and accept social support after release. Even when tangible support was available, this disruption may have reinforced avoidant behaviors. Indeed, prior research indicates that mass trauma is linked to decreased perceived social support, particularly among children and adolescents who rely heavily on their environment for stability (Aba et al., 2019). At the same time, short-term avoidant strategies may serve an adaptive role, facilitating a gradual transition from the stark isolation of captivity to the overwhelming sensory stimuli of reintegration (Stein et al., 2015)

Another form of avoidance was the reluctance or refusal to receive distressing news, with many adolescents trying actively to evade information about personal losses. Consistent with prior research (Hopwood and Schutte, 2017), our findings indicate that exposure to disaster-related media is associated with heightened anxiety and sleep disturbances. Vulnerability to such exposure is particularly relevant in the context of captivity, where hostages are deprived of external information and remain unaware of significant events. These findings highlight the need for a structured, gradual, and clinically monitored approach to media exposure, particularly regarding distressing information about the loss of loved ones. To be sure, regulating media exposure after release from captivity presents unique challenges, given the intense public and media attention surrounding high-profile hostage cases (Fletcher, 1996). On the other hand, for adolescents, social media serves as a primary means of communication and social connectedness and can provide benefits, such as increased perceived social support (Best et al., 2014). Given this developmental context, clinicians should implement tailored strategies to help adolescents navigate media exposure in a way that safeguards their psychological wellbeing.

### 4.3 Adult caregivers

Caregivers exhibited many of the same symptoms observed in adolescents, including hyperarousal, sleep disturbances, and excessive sharing of traumatic material. However, they also displayed dissociative symptoms, which appeared to function as coping mechanisms for managing the anguish of captivity. From a medical perspective, dissociation is defined as a disruption—acute or gradual, transient or persistent—of consciousness, perception, memory or awareness (American Psychiatric Association, 2013). Dissociative responses are often linked to maladaptive coping strategies and an elevated risk of post-traumatic symptoms (Lam and Fung, 2024). However, from a psychoanalytic perspective, certain forms of dissociation are considered defense mechanisms that can provide psychological elasticity, transforming the experience to protect the individual from psychic pain and to foster resilience (Alayarian, 2019). The caregivers in our sample had to maintain their parental role throughout captivity to protect their children. Upon return to a safe environment, however, they could temporarily “let go” of that role. We hypothesize that this transition opened up “psychic space” to process their experiences and grieve their losses. This shift may have resulted in moments of dissociation, serving as an adaptive response to overwhelming emotions.

### 4.4 Cross-age considerations

Significantly, practitioners consistently reported that children's reactions were transient, with many resolving within several days through appropriate intervention and the passage of time in a secure environment (the hospital). These findings highlight children's remarkable adaptability; as such, practitioners cautioned against pathologizing normal responses to an extremely abnormal event. While heightened reactions are common in the months following a highly traumatic event, only a small percentage of children develop long-term psychopathology (Bonanno et al., 2011). Although some may face genuine psychological challenges or struggle with post-incident adjustment, it is essential to shift from a pathogenic model to a resilience model that recognizes the extraordinary strengths that children and their caregivers often demonstrate in the face of trauma (Aba et al., 2019).

At the same time, this study was conducted in the immediate aftermath of captivity—a period often marked by euphoria, optimism, and an apparent return to normalcy, during which survivors may believe that their release resolves all difficulties (Fletcher, 1996). These powerful emotions can sometimes delay or prevent help-seeking behaviors. Nevertheless, it remains crucial to provide survivors and caregivers with information about potential psychological challenges and to guide them through the complexities of post-captivity life.

Two prevalent reactions spanning all age groups were sleep disturbances and the extensive sharing of traumatic material. Among these, sleep disturbances were the most widespread, underscoring the central role of sleep structure in revealing the effects of trauma. It has been suggested that trauma-associated sleep disorder (TASD) may constitute a distinct parasomnia—a specific sleep disturbance specifically linked to traumatic exposure (Brock et al., 2019). Notably, this condition is closely associated with hyperarousal, another prominent reaction observed across all age-groups in our study. While evidence supporting pharmacological and non-pharmacological treatments for TASD remains limited, improving sleep duration and quality through sleep hygiene—such as maintaining a consistent daily routine and minimizing stimuli before bedtime—presents a potential intervention for mitigating trauma-related sleep disturbances (Brock et al., 2019).

Another significant reaction observed among adolescents and caregivers was the extensive sharing of traumatic material. In some cases, this intense verbalization was surprising, even overwhelming, raising concerns among practitioners about emotional flooding. Excessive talking can manifest in response to the intense psychological and emotional turmoil following captivity (Fletcher, 1996). Although sharing experiences is a crucial and healthy coping mechanism following traumatic experiences and prolonged isolation, excessive or unstructured disclosure can become overwhelming and trigger overly strong emotional reactions (Rimé et al., 2010). Practitioners in our study responded by attempting to provide private spaces for sharing traumatic experiences in a safe, structured manner. In line with previous recommendations (McNally et al., 2003), this included providing a non-intrusive presence during family conversations (if requested), attentively listening to shared experiences while monitoring emotional reactions, and offering strategies for support and regulation.

The circumstances of captivity—notably, whether children, adolescents and caregivers were held together as a family or separately—was an important factor influencing post-release psychological responses across all age groups. This factor significantly shaped the presentation of reactions in the immediate recovery phase. Scheeringa and Zeanah (2001) emphasize the importance of viewing traumatic events and associated symptoms among both caregivers and children within the relational model of PTSD. According to this model, children are not only affected *directly* by exposure to traumatic events but are also affected *vicariously* by the trauma's influence on their parents' mental health along with the resulting disruptions in the parent-child relationship.

In our sample of young children, the caregiver-child relationship remained largely intact during captivity, with caregivers generally managing to meet their children's emotional and practical needs despite significant captivity-related constraints; conversely, maintaining caregiving roles served as a protective factor for the caregivers themselves. This model underscores the mutual benefits of the child-caregiver relationship in captivity, highlighting the importance, post-release, of preserving or reconstructing this bond.

This study has several limitations that should be considered when interpreting its findings and implications. Although the study drew upon multiple data sources—including qualitative interviews and medical records—to assess the reactions of children and caregivers, it did not collect direct accounts from the target population itself (i.e., the children and caregivers released from captivity). This methodological choice was made to prioritize the wellbeing of the children and their families during a highly vulnerable time. Additionally, the study was cross-sectional rather than longitudinal, thereby limiting the possibility of drawing conclusions about the trajectory and persistence of mental health difficulties over time. Future research is needed to ascertain the long-term psychological outcomes of captivity among both children and caregivers. Despite its limitations, however, the current study makes a valuable contribution to the understanding of the immediate impact of extreme trauma on children and caregivers' mental health and provides insight into practitioners' experiences during this complex intervention.

## 5 Conclusion and implications for practice policy and research

This study is the first to present qualitative data on the immediate psychological responses of children and their caregivers following release from war-related captivity. To help advance future research as well as improve policies and clinical practice we offer the following recommendations.

*Train frontline practitioners in trauma-informed care*. Practitioners must be specifically trained to recognize and respond to developmentally appropriate psychological reactions in the immediate aftermath of captivity. This training should be informed by PFA and consider the unique needs of both children and their caregivers.

*Conduct multidisciplinary assessments upon release from captivity*. Upon release, children and their caregivers should undergo comprehensive medical, psychological social and nutritional evaluations to assess both immediate and long-term mental health needs. These initial assessments provide healthcare teams with vital information, allowing them to develop a clear understanding of children's needs and to tailor interventions accordingly.

*Adapt and implement Psychological First Aid (PFA) principles across age groups*. Interventions should be grounded in the principles of the PFA model, adapted to the developmental stage of the child. Key strategies to promote emotional regulation and recovery in children include psychoeducation, cognitive reframing and organization, and the rapid establishment of structured behavioral routines. For adolescents and adults, supportive listening techniques should be employed, along with efforts to manage exposure to overwhelming traumatic content (e.g., via social media) (see {author's own} for in-depth guidance). It is critical that practitioners recognize immediate post-crisis reactions as normal and expected, avoiding the pathologization of these responses. Practitioners should also provide clear psychoeducation to caregivers, reinforcing that such reactions are typical responses to an abnormal and extreme event.

*Preserve and strengthen the caregiver-child relationship post-release and prioritize a family-centered approach to intervention*. In line with the relational model of PTSD, interventions should assess and reinforce the caregiving relationship as a critical component of the child's recovery. If children were held in captivity alone, efforts should be made to reunite them with existing family or community members, rebuild relational trust, and restore caregiving roles as a foundation for healing

*Offer time-limited, intensive early intervention*. Based on our findings that initial reactions tend to attenuate over time, post-release care should be intensive but limited in duration (one to three weeks), unless severe psychiatric or physical conditions are present. A balance must be struck between creating a safe space to begin the adjustment and healing process and encouraging a return to a home or home-like environment. Discharge decisions should be conducted collaboratively with families, carefully considering individual circumstances and the achievement of essential therapeutic goals.

*Ensure continuity of care and long-term support* release marks only the beginning of recovery. It must be followed by immediate and sustained psychological care, social reintegration, and structured rehabilitation efforts that are tailored to the unique experiences of abducted children and their caregivers. Long-term follow-up in a specialized trauma center is essential to ensure early detection and treatment of medical and mental health issues.

From a research perspective, our results identify key areas for future investigation that could help validate the studies preliminary observations. For instance, the seemingly adaptive role of dissociation among adults, and the complex, potentially both protective and harmful effects of social media among adolescents, warrant further longitudinal study to examine these effects over time. Moreover, given the nascent status of research focused on children abducted during armed conflict—and the wide variability in experiences and contexts—it is essential to conduct follow up studies that adapt interventions identified in this study to different conflict contexts.

## Data Availability

The datasets presented in this article are not readily available due to the very small sample size and the sensitivity of the data. Requests to access the datasets should be directed to mayafennig@tauex.tau.ac.il.
