# Correction: Early psychological responses of children and caregivers in the immediate aftermath of release from war captivity

**DOI:** 10.3389/fpsyg.2025.1676420

**Published:** 2025-08-13

**Authors:** Maya Fennig, Avigal Snir, Maayan Shorer, Efrat Bron Harlev, Silvana Fennig

**Affiliations:** ^1^The Bob Shapell School of Social Work, Tel Aviv University, Tel Aviv, Israel; ^2^School of Behavioral Sciences, Academic College Tel Aviv-Yaffo, Tel Aviv, Israel; ^3^Psychological Medicine Department, Schneider Children's Medical Center, Petach Tikva, Israel; ^4^Clinical Psychology Department, Faculty of Social & Community Sciences, Ruppin Academic Center, Emek Hefer, Israel; ^5^Gray Faculty of Medical & Health Sciences, Tel Aviv University, Tel Aviv, Israel

**Keywords:** children, trauma, war, Israel, conflict, mental health, adolescents, psychological intervention

In the abstract, some information was reported incorrectly in the Objectives and Methods:

Objective: This study aims to (1) describe the early psychological responses of children and caregivers' early psychological responses immediately following their release from captivity in the Israeli-Hamas war

Methods: This qualitative study analyzed the psychological reactions of children and their caregivers (N = 26) who were released from captivity and received care at {masked}

The correct sentences appear below:

**Objective:** This study aims to (1) describe children and caregivers' early psychological responses immediately following their release from captivity in the Israeli-Hamas war and (2) examine the clinical interventions used to manage these reactions.

**Methods:** This qualitative study analyzed the psychological reactions of children and their caregivers (*N* = 26) who were released from captivity and received care at Schneider Children's Medical Centre of Israel.

A correction has been made to the **Methods** section, subsection *Context and setting*, paragraph 3. The name of the institution previously included a [masked] placeholder. The correct sentence appears below:

“Conducted over a 4-month period (December 2023–March 2024), the study was a partnership between Tel Aviv University and Schneider Children's Medical Centre of Israel.”

A correction has been made to the **Methods** section, subsection *Study sample*, paragraph 1. The name of the institution previously included a [masked] placeholder. The correct sentence appears below:

“Table 1 displays the characteristics of the sample of children and their caregivers (*N* = 26) who had been abducted in the context of war and subsequently received treatment at Schneider Children's Medical Centre of Israel, where the study was conducted.”

A correction has been made to the **Methods** section, subsection *Study sample*, paragraph 2. The name of the institution previously included a [masked] placeholder. The correct sentence appears below:

“The study sample comprised a subset of 37 practitioners employed at Schneider Children's Medical Centre of Israel, including social workers (*n* = 11), psychologists (*n* = 10), a child psychiatrist (*n* = 1), nurses (*n* = 9), pediatricians (*n* = 4), and senior directors (*n* = 2).”

A correction has been made to the **Methods** section, subsection *Ethical considerations*, paragraph 1. The name of the institution previously included a [masked] placeholder. The correct sentence appears below:

“All procedures were reviewed and approved by the Institutional Review Board of Tel Aviv University and the Institutional Review Board of Schneider Children's Medical Centre of Israel.”

The original version of this article has been updated.

